# neXtA_5_: accelerating annotation of articles via automated approaches in neXtProt

**DOI:** 10.1093/database/baw098

**Published:** 2016-07-02

**Authors:** Luc Mottin, Julien Gobeill, Emilie Pasche, Pierre-André Michel, Isabelle Cusin, Pascale Gaudet, Patrick Ruch

**Affiliations:** ^1^BiTeM Group, University of Applied Sciences, Western Switzerland-HEG Genève, Information Science Department; ^2^SIB Text Mining, Swiss Institute of Bioinformatics; ^3^Calipho Group, Swiss Institute of Bioinformatics

## Abstract

The rapid increase in the number of published articles poses a challenge for curated databases to remain up-to-date. To help the scientific community and database curators deal with this issue, we have developed an application, neXtA_5_, which prioritizes the literature for specific curation requirements. Our system, neXtA_5_, is a curation service composed of three main elements. The first component is a named-entity recognition module, which annotates MEDLINE over some predefined axes. This report focuses on three axes: Diseases, the Molecular Function and Biological Process sub-ontologies of the Gene Ontology (GO). The automatic annotations are then stored in a local database, BioMed, for each annotation axis. Additional entities such as species and chemical compounds are also identified. The second component is an existing search engine, which retrieves the most relevant MEDLINE records for any given query. The third component uses the content of BioMed to generate an axis-specific ranking, which takes into account the density of named-entities as stored in the Biomed database. The two ranked lists are ultimately merged using a linear combination, which has been specifically tuned to support the annotation of each axis. The fine-tuning of the coefficients is formally reported for each axis-driven search. Compared with PubMed, which is the system used by most curators, the improvement is the following: +231% for Diseases, +236% for Molecular Functions and +3153% for Biological Process when measuring the precision of the top-returned PMID (P0 or mean reciprocal rank). The current search methods significantly improve the search effectiveness of curators for three important curation axes. Further experiments are being performed to extend the curation types, in particular protein–protein interactions, which require specific relationship extraction capabilities. In parallel, user-friendly interfaces powered with a set of JSON web services are currently being implemented into the neXtProt annotation pipeline.

**Available on:** http://babar.unige.ch:8082/neXtA5

**Database URL:** http://babar.unige.ch:8082/neXtA5/fetcher.jsp

## Introduction

Over the past decades, biomedical literature has grown exponentially (1). In parallel, the research community needs to have access to data in computable forms to support powerful querying and complex data analysis. Meanwhile, most knowledge acquisition methods in biocuration are based on manual approaches. Few automatic methods are in use in real-life settings. The process is thus labour intensive and time-consuming (2, 3). The development of effective methods to automatically process the literature promises to make increase the efficiency of the curation process, thus enabling to parse a much larger fraction of the literature.

To balance the need of efficiency and resources limitations with the expansion of the literature, text-mining tools such as document-triage engines (or prioritization engines) and named-entity recognizers (that recognize and highlight specific entities in texts) have been designed. These tools can be integrated at different stages of the curation process (3–5): triage, indexing, extraction of evidences, validation and recording. Among the numerous software developed in recent years (6–14), the most performant are usually quite specific, focusing on a narrow scope (6, 10) or a given ontology (7, 11). However, most of these systems usually do not offer a fully integrated workflow, displaying prioritized abstracts together with proposed annotations, for the validation by the biocurators and thus, ready for database insertion.

neXtA_5_ is an application developed to support biocuration activities at neXtProt (15). The goal of this project is to create an annotation workflow that will leverage automated methods for text analysis to speed up curation. The final system should retrieve pertinent documents across MEDLINE, mine them with specific ontology-driven named-entity recognition systems and generate annotations. The current project is an active collaboration with the neXtProt database (16), whose main outcome is a system operated via publicly accessible Application Programming Interface (APIs) and web services. These tools will then be integrated into a Swiss Institute of Bioinformatics (SIB) tool called Bioeditor (17), as well as other curation workflows.

In this report, we evaluate a specific component of the workflow, neXtA_5_, which aims to improve the triage function for some of the neXtProt curation axes. In this article, we focus on three types of annotation: Diseases, Molecular Functions (MFs) and Biological Processes (BPs). Diseases are defined as National Cancer Institute thesaurus (NCIt) concepts, while MFs and BPs are defined as Gene Ontology (GO) concepts.

## Materials and Methods

In this section, we introduce the resources used by neXtA_5_, including the terminological resources we defined and the benchmarks we generated to assess the ranking power of the system. Then, we describe the neXtA_5_ ranking functions, which we evaluated for each of the three annotation axes.

### Diseases

The vocabulary chosen for Diseases annotation is the NCIt (18–20), 7 November 2014 release, obtained from https://ncit.nci.nih.gov/ncitbrowser in the Web Ontology Language format. For each concept, we restricted the fields to preferred terms, synonyms (SY) and abbreviations. However, this version is enhanced by cross-linking different SY form the medical subject heading (MeSH) vocabulary, using the unified medical language system. The resulting vocabulary is slightly richer than the original NCIt. From this controlled vocabulary, we also filtered out the most frequently used English words and words with a length shorter than four characters (21). This step aims at removing false positives caused by entries such as ‘CAN’, an acronym for ‘Chronic Allograft Nephropathy’ (NCIt ID: C38145).

### Gene Ontology

The GO is a vocabulary designed to describe the role of genes and gene products across all species (22, 23). This resource is subdivided in three axes: the MF describes molecular activities of proteins, while the BP captures how these molecular activities are connected to perform coordinated events (e.g. DNA replication).The cellular component (CC) characterizes physical locations (organelles or complexes) within the cell or the extracellular environment. Compared with MF and BP, the CC have not extensively been annotated in neXtProt, therefore this study focuses on MF and BP. In GO, each entity is defined with a unique alphanumeric identifier, a term name, a definition and its axis. SY may be associated to the term with their related relationship as represented in Table 1. The version of GO from 25 November 2014 was used for this work and regular updates (every month) are planned at exploitation time. We tested the SY of different types (EXACT, NARROW and RELATED) with little effect on the performance of the system.

**Table 1.** Example of GO BP entity with valid SY

[Term] id: GO:0030318 name: melanocyte differentiation namespace: biological_process def: “The process in which a relatively unspecialized cell acquires specialized features of a melanocyte.” [GOC:mah] synonym: “melanocyte cell differentiation” EXACT [] synonym: “melanophore differentiation” EXACT [] is_a: GO:0050931! pigment cell differentiation is_a: GO:0060563! neuroepithelial cell differentiation

### Additional terminological resources

Together with the three curation axes described in the previous sections, several entities are also identified in the body of the abstracts and titles. Then, entities such as species [National Center for Biotechnology Information (NCBI), taxon identifier], chemical compounds Chemical Entities of Biological Interest (ChEBI) and evidence codes (ECOs) are also identified although they are not currently used to compute the ranking functions as they do not constitute a curation axis. However, these entities are highlighted at curation time to ease the reading of the abstracts. Regarding the species, we are working on a subset of specimens identified by their TaxID form the NCBI. The list covers the curation needs of neXtProt. Species are mined in texts with their official and common names.

The ChEBI is a dictionary based on chemicals molecules available on http://www.ebi.ac.uk/chebi/init.do. We extracted only information about short and developed name, besides their accession number. Finally, we use the Evidence Ontology (ECO), 26 January 2015 release, covering laboratory experiments and computational methods. A subset of the original ECO nomenclature is combined with ∼30 new codes proposed by the curators. The terminology distributions are shown in Table 2.

**Table 2.** Index size per axis after thesauri refinement

Thesaurus	#terms
Diseases	97545
GO MF	36068
GO BP	99242
GO CC	6459
ECO	174
Species	81
ChEBI	41

### Triage

Triage can be performed using a wide span of methods. It is thus possible to envisage the work as a machine-learning task. In that case, the triage system is trying to classify a given article into some inferred categories. The classification can be binary (relevant/non-relevant) or multiclass (relevant for a particular annotation axis). Classification framework, such as support vector machine, neural networks or naïve Bayes, are basically binary classifiers, which can be used to perform multi-class classification problems as in Wormbase (24). In the past, Text Retrieval Conference (TREC) Genomics has for instance evaluated triage as a classification problem to support the annotation of the Mouse Genome Database with inconclusive results: no system was able to beat the baseline, whose strategy was to select articles based on MeSH descriptors assigned by librarians of the National Library of Medicine. The main challenge was clearly the acquisition of negative training data since the ‘negative data gap’—database managers do not keep track of articles regarded as irrelevant by curators—is a well identified shortcoming of today’s data stewardship (25). The automatic acquisition of negative samples is possible but challenging (26), therefore neXtA_5_ is designed as a learning to rank system (27).

Triage can indeed be opportunely considered as a ranking problem, as explored by BioCreative 2012, Track I (28)—to support the annotation of the Comparative Toxicogenomics Database (29). This option seems more suitable when designing an interactive system, as it does not attempt to make the triage decision, but instead it only attempts to display the most relevant articles on the top of the list.

The separation between classification and ranking is convenient but relative as it is always possible to transform a binary classifier into a ranking system, for instance by using the class probability as an analogue of a similarity measure. Symmetrically, a ranking system can be turned into a classifier by setting an empirical boundary threshold. However, a ranking model can be regarded as more flexible and more appropriate to the knowledge extraction workflow of neXtProt as the curators are used to query retrieval engines. It has also the advantage to be able to gracefully operate with sparse positive and no negative tuning data.

### neXtA_5_ design

**Figure 1.**
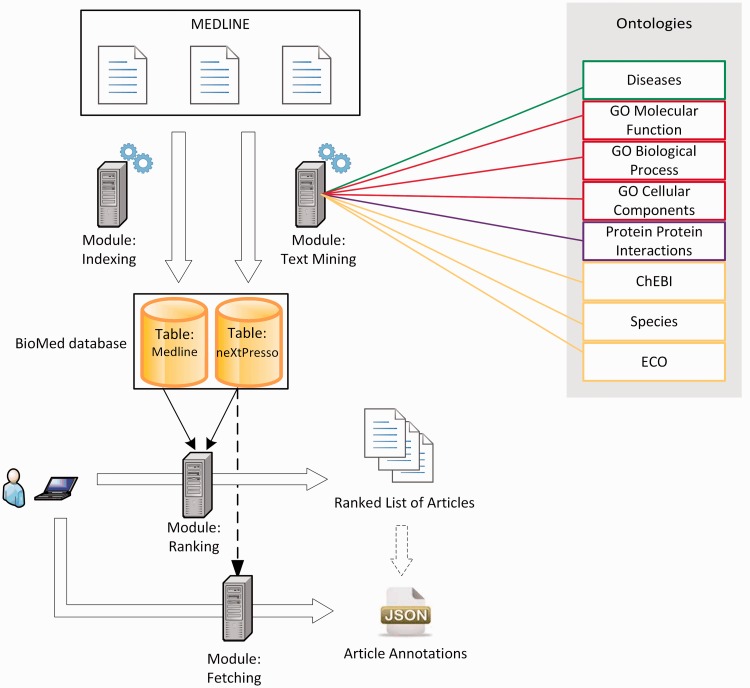
The neXtA_5_ functional architecture.

Inspired by ToxiCat (30), which achieved competitive results during BioCreative 2012, neXtA_5_ is a java/javascript-based platform intended to enhance the curation of MEDLINE. Figure 1 shows the functional services of neXtA_5_. The input of the ranking module is a triplet {protein—research mode—axis}, the output is a ranked list of PMIDs, where bio-entities have been normalized.

The automatic identification of entities, available through these aforementioned terminological resources, is performed on the whole MEDLINE *a priori* to speed up user interaction with the system. The only task performed at query time is the ranking of documents.

The preprocessing steps include a text-mining module that automatically extracts and annotates a local copy of MEDLINE, synchronized on a weekly basis. Articles are processed individually, vocabulary by vocabulary, matching entities in both titles and abstracts and starting with the longest descriptors. This way, the system is likely to capture more specific entities. Thus, ‘Papillary Breast Cancer’ (ID C9134 in NCIt) will be preferred over ‘Breast Cancer’ (ID C4872), which will be preferred over ‘Cancer’ (ID C9305). Table 2 presents the number of terms contained in the vocabulary, together with three complementary vocabularies: for chemical entities (ChEBI), species and other evidences (ECO). All named-entities found in MEDLINE are stored in a local database within tuples such as {PMID; Term ID; Term Frequency (TF); Textual Form}. ‘Term ID’ allows neXtA_5_ to associate SY while ‘Textual Form’ corresponds to the string as found in the abstract or the title.

Our information retrieval (IR) component proposes two research modes; see Gobeill *et al.* (31, 32) for a detailed presentation of the process and settings of the engine. The first mode makes possible to perform Boolean queries directly using PubMed via the e-Utils. In this case, the search engine score assigned to each PMID is ∼0 and the rank is strictly obtained from PubMed. The second is a search engine based on a vector-space model that locally indexes the content of MEDLINE (33). This mode is referred to as ‘vectorial’ mode. A specific weighting schema (combining Okapi BM25 and Deviation from randomness) has been tuned (34, 35).

Finally, the ranking function is based on a linear combination of factors (36, 37): the search engine score, the number of distinct matched terms (matching index size) and the TF. These three elements were experimentally weighted, and we adjusted the coefficients to obtain an optimal axis-specific scoring function. A post-ranking filter is applied on the retrieved documents to exclude ‘retracted articles’ and ‘reviews’, two MEDLINE article types that should be excluded from the curation process.

**Figure 2.**
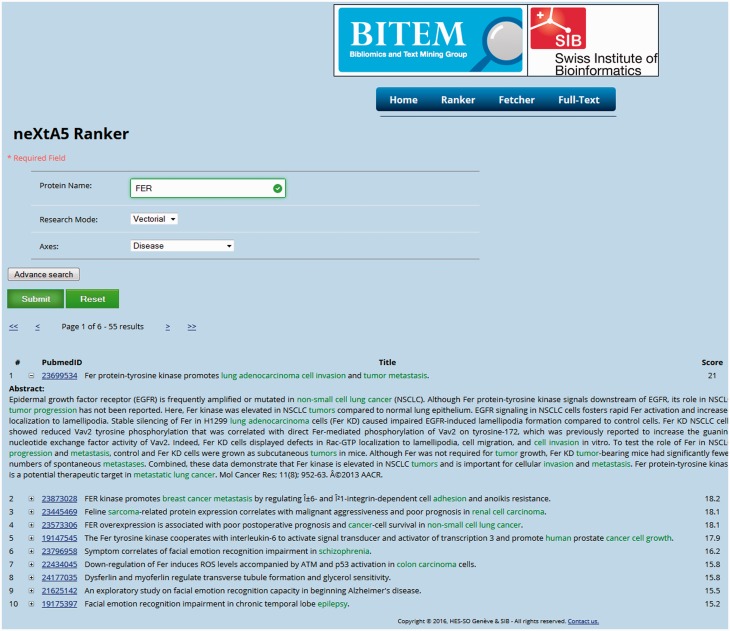
The neXtA_5_ web interface. The query was {FER—vectorial—Diseases}, with 1990 as the lower limit (the date can be modified in advanced mode) for the publication dates. The output presents the first fifty results ranked over the chosen axis, with the score of the linear combination and the concepts identified in the PMID.

The prototype interface presented in Figure 2 is designed to clearly display the results of the ranking function as an ordered list of PMIDs. The minimal input of the application requires a user query (e.g. a protein), an annotation axis and a retrieval mode (Boolean or vectorial). An advanced mode allows performing a more precise request by adding filters on publication dates or gene SY, which can be accessed using the UniProt accession numbers, to expand the query. Finally, a fetching interface provides the list of concepts harvested by neXtA_5_ for a given PMID. The web service can access the content of BioMed and supports an output in a JavaScript Object Notation (JSON) format, as shown in Table 3.

**Table 3.** JSON annotation example for PMID: 23883606^a^

{“**error**”: ““, “**pmid**”: “23883606”, “**result**”: [{“**termid**”: “C26740”, “**tf**”: 1, “**form**”: “dehydration”}, {“**termid**”: “GO:0008900”, “**tf**”: 1, “**form**”: “gastric h/k atpase”}, {“**termid**”: “GO:0008900”, “**tf**”: 1, “**form**”: “gastric h+/k+ atpase”}, {“**termid**”: “GO:0016020”, “**tf**”: 3, “**form**”: “membrane”}]}

^a^This article contains six concepts: one from NCIt (“dehydration”) and three GO (two different synonyms for “hydrogen:potassium-exchanging ATPase activity” and three occurences of the term “membrane”). Bold entries represent the JSON fields that could be captured from the web service.

### Evaluation and benchmark description

Every year since 1992, the TREC organizes competitions to assess advances in IR. Hence, the TREC methodology has been used to generate various reference benchmarks. A more detailed presentation of TREC standards and metrics, which are used in this report, can be found in Singhal 2001 (38). The formal benchmark is composed of three different data sets: (i) a set of queries, (ii) a corpus of documents and (iii) a set of relevance judgements, which links each query to one or more documents considered as relevant.

In IR, the precision and the recall are the metrics consensually adopted to establish to what degree the system obtained the expected results (39). The recall represents the fraction of the relevant documents set that are retrieved by a given query, whereas precision is the proportion of documents retrieved by the system that are relevant. In large collections, the recall can only be estimated on a relative scale, therefore IR is mainly measured with respect to the precision. Because relevant documents returned at high ranks are more useful, evaluation is measure at different ranks between 0 and *n* and P0 (the precision at Rank #1) is regarded as the most stringent measure.

To construct our reference benchmark, also called QREL (Query Relevance document), we used annotations supplied by neXtProt for 100 kinases. These one are extracted from a 2-years manual work involving 14 curators, Table 4 displays the proportions among focused axes. Then, we ran the TREC evaluation tool (trec_eval) to compare our QREL with the results provided by neXtA5 (with different settings) on the same kinases (Supplementary data). Strictly in the same way, we evaluated the results provided by PUBMED on these queries. As experimented with TREC and BioCreative evaluations when they operate with curated data (Mouse Genome Database, Gene Ontology Annotation, IntAct…), the quality of the annotation is taken as ‘ground truth’. Such an assumption is relatively weak if we consider that curated data reflect as state of the knowledge at a given time. Perfect benchmarks do not exist neither regarding coverage nor stability.

**Table 4.** Distribution of annotated proteins and PMIDs in the benchmark for each annotation axis

Thesaurus	#kinases	#terms
Diseases	100	4839
GO MF	18	47
GO BP	100	3189

Even if there are articles that were omitted by the curators, we estimate that curated data provide a good estimate of what should be captured by an automatic system. Finally, as the purpose is not only to retrieve the exact same list of papers, but also to rank the most relevant at the top, our evaluation represents a fair—yet relative—evaluation of the different components and settings of a triage system.

With the objective to enhance mainly the precision, we evaluated the performance of neXtA_5_ by tuning 1042 combinations of parameters, see section ‘neXtA_5_ Design’. We compared our results against two systems (as baselines): the vectorial search engine, which is a strong baseline, and the ‘Relevance’ version of PubMed, which is the typical tool used by curators.

## Results

**Figure 3.**
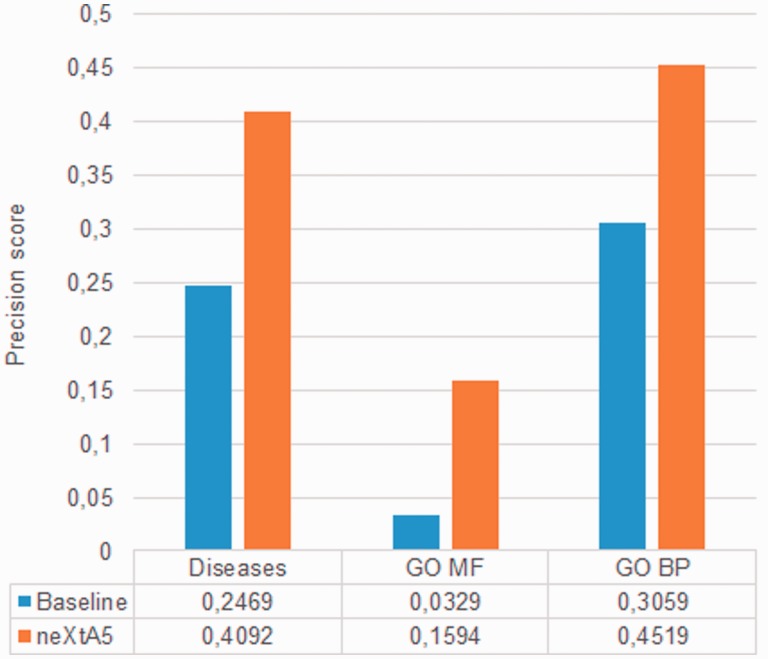
Optimization of the ranking function for each axis.

We first establish a strong baseline by evaluating the search effectiveness of the vector-space search mode (40–42). Figure 3 represents the results obtained for the three evaluated axes at the top-returned PMID (P0). The blue histograms show the performances of the vectorial search engine, while the red histograms show the precision affected by tuning. Thus, we observe a gain of +66%, +48% and +385% for Diseases, BP and MF axes, respectively. The experimental improvement is statistically significant for all axes (*P* > 0.05). The gain for the Diseases axis reaches +66% (from 25 to 41%), which means that the top ranked PMID in neXtA_5_ is a relevant one more than four times out of 10 compared with approximately one time out of four in the baseline system.

The second most significant improvement is observed for BP with an increase of +48%. The gains for MF, with almost +385%, is even higher, however such an improvement could be imputable to the limited number of relevant documents in the curators’ benchmark (Table 4). Thus, using the current parameters, neXtA_5_ has different impact on curation with respect to the axis with improvements ranging from ∼50% to nearly 400%. The linear combinations providing the optimal ranking functions are the following:

Diseases: *Article score = 1.0 × search engine score + 0.5 × matching index size*.
BP: *Article score = 0.9 × search engine score + 1.0 × matching index size*.
MF: *Article score = 1.5 × search engine score + 0.3 × term frequency*.

Future versions of the weighting schemas should clearly take into account usage patterns, and therefore these coefficients are likely to vary in the future. Yet, the choice to design axis-driven search strategies, as opposed to creating a general purpose curation engine, is fully validated.

### Comparison with PubMed

The reference search platform for biocuration is PubMed, the NCBI search engine on the top of the MEDLINE digital library. PubMed offers different ranking functions. The default one is a Boolean query engine that sorts results in reverse chronological order, with most recent publications on top. Otherwise, the ‘Relevance’ mode sorts citations according to statistical measures (43). In this mode, priority is still given to recent articles.

**Figure 4.**
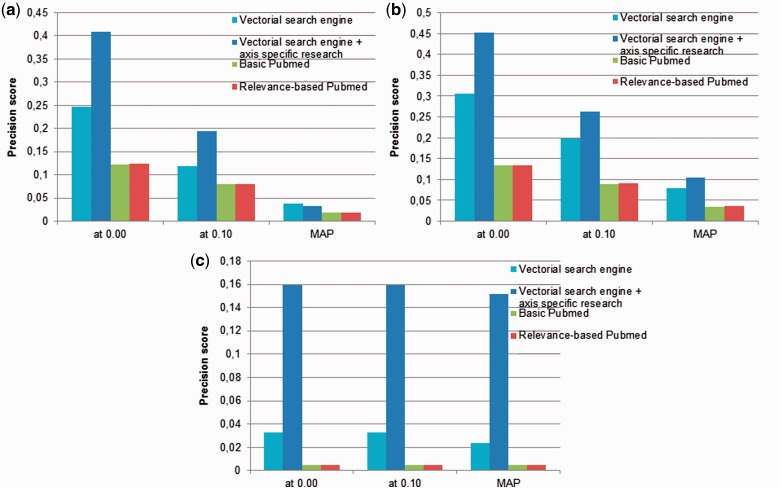
Precision (at P0, P10 and MAP) obtained by the neXtA_5_ vector-space retrieval model compared with the PubMed search modes: (**a**) Diseases, (**b**) BP and (**c**) MF.

In Figure 4, we compare the performances of the neXtA_5_ ranking function with the ranking proposed by PubMed on the BP and Diseases annotation axes. The two PubMed ranking modalities are examined: the default method and the so-called ‘Relevance’ sorting. Regarding search, the differences between the two models are marginal. For these systems, we also measure the mean average precision (MAP), which computes the average precision at different ranks (top-10, top-20, etc.). If we consider, for instance, the Diseases axis, the two search modes obtain extremely close results regarding MAP. Regarding the top precision, the Relevance-based mode performs slightly better than the default one (0.124 vs. 0.122 → +1.6%). In contrast, neXtA_5_ obtains a top precision of 0.41. Thus, compared with the best retrieval strategy of PubMed, which is likely to be used as default by many curators, the relative gain shown by the neXtA_5_ is approximately +108% on the MAP. Similar improvements are reported for BP and MF with, respectively, +192% and +2998%.

At P0 (interpolated precision at recall = 0.00), the best improvement to support a Diseases curation task is +231%, whereas it is respectively of +236% and +3153% for BP and MF curation when PubMed ‘Relevance’ is used as baseline system.

## Discussion

Although the comparison against PubMed is obviously in favour of the neXtA_5_ ranking algorithm, the reported results vary significantly with the considered axes. Further, when we pay attention to precision at top ranks, the improvement is impressive with a maximum gain of +3153%, obtained for MF.

Assessing the absolute advantage of the system is difficult and evaluations based on utility metrics (e.g. processing time, quality improvement…) will be needed, however it is worth observing two important aspects. The neXtProt data used as benchmark in our experiments should not be regarded as perfect because of its coverage. Although the quality of the curation defines our gold standards, the usual curation process is protein-centric rather than paper-centric. Most of what is known about a given protein at a given time is likely captured by the neXtProt curators. Nevertheless, we are aware that covering all published—and often redundant—papers is obviously not a goal for curators. Our experimental measures are directly derived from the neXtProt benchmark, which has a particular creation date. Therefore, the outputs of our search engines have been adapted to limit the date of the returned documents (1990–2013).

In the same vein, the current system is taking only MEDLINE contents as input, while the triage performed by curators may have taken into account additional contents such as full-text articles (44), including figures, tables or supplementary files that were not available for neXtA_5_. It is expected that working with these additional data may have improved our results; however, this comparison is fair regarding our baseline search engines.

## Conclusion

We presented in this report, neXtA_5_, a ranking service and interface embedded into a curation platform. This application is powered by various ontologies, which are used to provide a better ranking of MEDLINE articles for sake of curation and a vectorial search engine. When compared with the best PubMed search model, the precision (P0) of neXtA_5_ is, respectively, improved by +231%, +236% and +3153%, respectively, for the Diseases, the BP and the MF curation, after the accurate tuning of our local vector-space search engine.

We are now drafting more specific ranking functions to perform the curation of protein–protein interactions, and similarly we plan to further explore the MF. In particular, we intend to fix up our vector-space model, and to customize neXtA_5_ to support the curation using full-text articles in complement to abstracts.

## Supplementary Data

Supplementary data are available at *Database* Online.
